# Role of Inflammation in Muscle Homeostasis and Myogenesis

**DOI:** 10.1155/2015/805172

**Published:** 2015-10-05

**Authors:** Domiziana Costamagna, Paola Costelli, Maurilio Sampaolesi, Fabio Penna

**Affiliations:** ^1^Department of Clinical and Biological Sciences, University of Torino, 10125 Torino, Italy; ^2^Stem Cell Research Institute, University Hospital Gasthuisberg, 3000 Leuven, Belgium; ^3^Interuniversity Institute of Myology (IIM), Italy; ^4^Human Anatomy Section, University of Pavia, 27100 Pavia, Italy

## Abstract

Skeletal muscle mass is subject to rapid changes according to growth stimuli inducing both hypertrophy, through increased protein synthesis, and hyperplasia, activating the myogenic program. Muscle wasting, characteristic of several pathological states associated with local or systemic inflammation, has been for long considered to rely on the alteration of myofiber intracellular pathways regulated by both hormones and cytokines, eventually leading to impaired anabolism and increased protein breakdown. However, there are increasing evidences that even alterations of the myogenic/regenerative program play a role in the onset of muscle wasting, even though the precise mechanisms involved are far from being fully elucidated. The comprehension of the links potentially occurring between impaired myogenesis and increased catabolism would allow the definition of effective strategies aimed at counteracting muscle wasting. The first part of this review gives an overview of skeletal muscle intracellular pathways determining fiber size, while the second part considers the cells and the regulatory pathways involved in the myogenic program. In both parts are discussed the evidences supporting the role of inflammation in impairing muscle homeostasis and myogenesis, potentially determining muscle atrophy.

## 1. Introduction

Skeletal muscle is the most abundant tissue in human body, except in obese patients, and is involved in several physiological functions. Indeed, glucose uptake and metabolism take place primarily in the skeletal muscle, a tissue prone to adaptation in size by means of both hypertrophy and hyperplasia. The former relies on the regulation of protein synthesis and degradation rates, while the latter involves the myogenic process that is in charge of regulating myocyte turnover as well as of supporting the rapid regeneration following injury.

The counterpart of muscle hypertrophy is muscle atrophy, even defined as sarcopenia, that naturally occurs in physiological conditions, such as aging [1]. Beyond aging, muscle wasting is a feature associated with several pathological states and chronic diseases such as immobilization following fractures or bed rest, malnutrition, cancer, CHF, CKD, COPD, burns, muscular dystrophies, AIDS, sepsis, and immune disorders [2]. Muscle depletion has important implications, exercise intolerance and inability to manage daily activities that eventually translate into poor quality of life. Most of the above mentioned pathological conditions are associated with variable degrees of local and/or systemic chronic inflammation, an element that could play a relevant role in the onset of muscle wasting [2]. Indeed, inflammation is considered one of the diagnostic hallmarks of cachexia, a wasting condition that often occurs in chronic diseases [3].

The aim of this review is to summarize the evidences supporting the role of inflammation, associated with several illnesses, in impairing muscle homeostasis and myogenesis, leading to muscle atrophy.

## 2. Muscle Homeostasis, Atrophy and Hypertrophy Pathways

Skeletal muscle mass represents a determinant of physical performance, and muscle size varies according to physiological stimuli and pathological conditions that, in turn, modulate the activation state of signaling pathways involved in the control of protein turnover. Muscle atrophy occurs when the balance between protein degradation and protein synthesis is poised towards degradation, leading to the loss of myofibrillar proteins and, consequently, to reduced fiber cross section area, finally resulting in impaired contraction ability and low force generation. Muscle nitrogen balance is finely modulated by distinct agents, both intrinsic (nutrient and energy availability, mechanical stress) and extrinsic (humoral mediators: hormones and cytokines). Moreover, muscle wasting, beyond the loss of muscle mass, often determines a reduction of muscle quality, that is, specific force, as reported in patients with CHF [4] or cancer [5] or admitted to the intensive care unit [6].

Protein breakdown in the skeletal muscle is mediated by two main degradation systems, the ubiquitin-proteasome and the autophagy pathways. The proteasome system preferentially targets short-lived proteins, and several reports considered the proteasome as the degradation machinery mostly involved in wasting processes of distinct origin [7].

The autophagy system is in charge of degrading long-lived proteins and organelles (mitophagy, pexophagy, etc.), and recent observations suggest that, beyond the proteasome, even autophagy plays a crucial role in muscle wasting [8]. In addition to proteasome and autophagy dependent proteolysis, intact myofilaments were postulated to undergo a preliminary cleavage in order to be released from the myofibrils for the subsequent ubiquitin dependent degradation, and such activity was proposed to be carried out by calpains [9] or by caspase-3 [10].

The observations reported above prompted the idea that protein breakdown inhibition could be the right way to prevent disease-associated muscle wasting. However, directly targeting the different proteolytic systems is unlikely an effective strategy. Indeed, proteasome inhibition proved effective only in experimental muscle unloading [11], while several reports show the detrimental effects of autophagy suppression [12–14]. Reasonably, both defective and excessive autophagy are deleterious by opposite mechanisms, namely, the lack of damaged protein/organelle removal and the exaggerated degradation, respectively. A distinct strategy in order to reduce muscle protein breakdown would be to target the muscle-specific ubiquitin ligases, since their activity represents the limiting step in determining both substrate-specificity and degradation rate. Since the beginning of the 2000s, the discovery of muscle-specific E3 enzymes and the characterization of their substrates have emerged and are still growing. The first ones, MAFbx/atrogin-1 and MuRF1/TRIM63 [15, 16], are actually the most commonly used read-out measurements for the molecular assessment of muscle protein catabolism, even though obvious limitations are implied. Few years later, TRIM32 was shown to target myofibrillar components [17], but its role seems to be primarily related with muscular dystrophies rather than wasting processes. The role of FBXO40, firstly characterized in denervation-induced atrophy in 2007 [18], was then clearly established by the identification of IRS-1 as substrate, thus defining a negative feedback on the anabolic PI3K/Akt axis [19]. Finally, FBXO30/MUSA1 was described as a BMP-regulated gene required for denervation- and fasting-mediated muscle loss [20]. However, investigations in humans are still lacking and no evidence is actually available in order to validate the use of ubiquitin ligases as therapeutic targets for muscle wasting.

**Figure 1:**
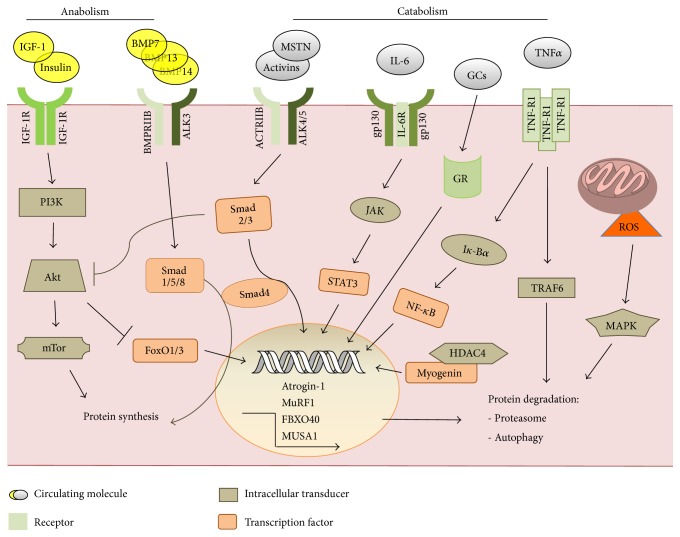
Humoral mediators and associated pathways drive anabolic and catabolic responses in the skeletal muscle.

Muscle protein degradation systems are modulated by a coordinated network of signaling pathways activated or suppressed by hormones and cytokines (Figure 1). On one hand, anabolic signals are activated by insulin, IGF-1, GH, and androgens, while catabolism is stimulated by a variety of proinflammatory cytokines as well as glucocorticoids and ROS. IGF-1 promotes muscle hypertrophy, while low IGF-1 circulating levels have been associated with several muscle atrophy conditions [21]. IGF-1, and similarly insulin, activates the PI3K/Akt pathway, which promotes protein synthesis through mTOR and its downstream effectors, mTORC1/2 complexes, not to mention that mTOR activation in the skeletal muscle results in autophagy inhibition [22], thus determining at the same time both protein synthesis activation and inhibition of protein degradation. Another IGF-1-mediated anticatabolic action is due to Akt phosphorylation/inhibition of the FoxO transcription factor family, determining their inability to translocate to the nucleus and promote the expression of the ubiquitin ligases atrogin-1 and MuRF1 [23] and autophagy genes [24].

Opposite to the IGF-1 pathway, one of the most relevant inducer of muscle atrophy is myostatin, a member of the TGF-*β* family. It signals through ACTRIIB, and recruiting the transcription factors Smad2/3 leads to increased atrogin-1 and MuRF1 mRNA levels [25]. The negative regulation of muscle mass exerted by myostatin likely relies on the suppression of Akt signaling [26].

In the complex network of signals relevant to muscle homeostasis, the BMP pathway has been recently characterized [20], showing that the downstream activation of the transcription factors Smad1/5/8 regulates a fundamental anabolic signal. In the same paper, the alternative activation of either the myostatin or the BMP pathway, both competing for Smad4 recruitment, was demonstrated. Indeed, BMP inhibition reverts the hypertrophic phenotype of myostatin-K.O. mice, suggesting that the balance between these signal cascades is crucial for the modulation of muscle mass.

Finally, few signaling pathways, less characterized in the skeletal muscle, displayed their relevance to muscle mass regulation during wasting conditions. Histone deacetylases 4 and 5 were shown to abrogate Dach2 expression that in turn lead to myogenin increase and ubiquitin ligase accumulation [27]. Overexpression of ATF4 was sufficient to induce muscle atrophy, regulating the expression of genes mainly related to cell growth suppression [28]. Similar to ATF4 action, the transcription factor and tumor suppressor p53 was able to trigger muscle atrophy through the induction of the cyclin-dependent kinase inhibitor p21 [29].

## 3. Inflammation Triggers Protein Catabolism and Impairs the Anabolic Response in the Skeletal Muscle

Loss of muscle mass, a common feature of chronic diseases, is frequently associated with increased production of proinflammatory cytokines such as TNF-*α*, IL-1, IL-6, and IFN-*γ*. For example, patients affected by chronic renal or heart failure show increased circulating levels of TNF-*α* and TNF soluble receptors [30, 31]. Similarly, several proinflammatory cytokines are increased in cachectic cancer patients (reviewed by [32]). Chronic inflammation may depend on increased expression of proinflammatory mediators but also on reduced levels of anti-inflammatory factors; consistently, mice K.O. for IL-10, one of the best known anti-inflammatory cytokines, display weakness and accelerated muscle loss [33] that can be improved by treatment with the anti-inflammatory, resveratrol rich, grape seed extract [34]. In addition to cytokines, other factors are produced during the inflammatory response, such as the so-called acute phase proteins, markers of systemic inflammation. In this regard, cancer cachexia and the acute phase response appear to be correlated: the enhanced synthesis of acute phase reactants in the liver has been proposed to drive muscle protein hypercatabolism contributing to increase of the resting energy expenditure [35]. Of interest, acute phase proteins have been shown to be produced even by the skeletal muscle itself [36].

Proinflammatory cytokines, together with altered homeostasis of classical hormones, put on a complex network that results in inhibition of anabolic and/or anticatabolic signals (see above), in favor of lipolysis and proteolysis. In particular, proinflammatory cytokines are well known to impinge on muscle protein metabolism. In this regard, data obtained in both experimental models and human pathology have demonstrated that systemic inflammation is associated with reduced rates of protein synthesis paralleled by enhanced protein breakdown, both accounting for the loss of muscle mass. However, the precise mechanism by which inflammation modulates protein turnover rates is still poorly investigated. Data obtained in clinical studies show a variegated situation: both rates are markedly increased in severely burned patients, with the balance remaining in favor of degradation [37]; synthesis rates are maintained in face of enhanced degradation rates in critically ill septic subjects [38], while in cancer patients with cachexia, protein turnover rates have been described as increased, decreased, or unchanged (reviewed in [39]).

The regulation of muscle protein metabolism by humoral factors is widely accepted. In this regard, the activity of both proteasome and lysosomes, the two proteolytic systems mainly involved in muscle depletion, is known to be affected by the hormonal and cytokine milieu. As an example, healthy animals exposed to proinflammatory cytokines such as TNF-*α*, IL-1, or IL-6 develop muscle wasting associated with increase of both ubiquitin expression and proteasome enzymatic activity (reviewed in [40]). Consistently, few studies demonstrated in the past that cytokines play a crucial role in the onset of muscle wasting. Indeed, muscle depletion, enhanced protein breakdown, and increased ubiquitin can be prevented by treating tumor-bearing animals with antibodies directed against IL-6, TNF-*α*, or IFN-*γ* [41–43]. Proinflammatory cytokines contribute to muscle wasting also in chronic diseases of noncancer origin. Indeed, increased circulating levels of TNF-*α*, IL-1, and IL-6 in sepsis appear to be correlated with disease severity and lethality. Similarly, the proinflammatory shift occurring in AIDS patients likely accounts for muscle protein hypercatabolism, a feature frequently reported in these patients before the adoption of combined antiretroviral therapy [44]. A recent report shows that muscle wasting in diabetic rats is associated with enhanced expression of TNF-*α*, IL-1, and IL-6 in the skeletal muscle and that such increase can be corrected by exercise training [45]. Finally, also sarcopenia and the loss of muscle quality that characterize aging are associated with high levels of proinflammatory mediators [46, 47].

The effects exerted by proinflammatory cytokines on muscle mass are mediated, partially at least, by activating the transcription factor NF-*κ*B. The transcriptional activity is regulated by the phosphorylation and consequent degradation of the inhibitor I*κ*-B*α*, allowing the positive regulation of MuRF1 [48] and other atrophy related genes, including the inducible nitric oxide synthase [49]. Studies performed on experimental models suggest that the NF-*κ*B signaling is activated in skeletal muscle during cancer cachexia, and recently modulations of this transcription factor have been observed in gastric and lung cancer patients [50, 51], as well as in experimental models [52]. Another protein related to the TNF-*α* cascade, TRAF6, was shown to coordinate the activation of both proteasome and autophagy [53].

Among proinflammatory cytokines, TWEAK has been shown to induce muscle wasting mainly by stimulating proteasome-dependent proteolysis [54]. TWEAK-induced downregulation of both PGC-1*α* expression and mitochondrial biogenesis has been proposed to mediate the effects on muscle mass. Indeed, PGC1*α* hyperexpression protects against TWEAK-induced effects such as NF-*κ*B activation, increased ubiquitin ligase levels, and muscle wasting [55]. In addition, several cytokines act on the JAK/STAT pathway, and muscle STAT3 was demonstrated to induce atrogin-1 [33] and to block autophagy, leading to muscle degeneration [34].

Proinflammatory cytokines act on muscle protein metabolism not only by activating catabolic pathways, but also by downregulating the anabolic ones. As an example, lipopolysaccharide-induced muscle wasting is associated with increased circulating levels of TNF-*α* and IL-1 that lead to inhibition of the Akt/mTOR signal transduction pathway [56, 57]. In this regard, treatments able to restore Akt physiological activity appear to counteract TNF*α*- induced wasting [58, 59]. Recent observations show that cancer cachexia occurring in the Apc (Min/+) mice also depends on the inhibition of mTOR activation due to the high circulating IL-6 levels [60]. The antianabolic action of proinflammatory cytokines is partially exerted through interactions with the IGF-1-dependent signaling pathway. Indeed, TNF-*α* leads to serine phosphorylation of IRS-1, inhibiting its recruitment to the insulin/IGF-1 receptor. TNF-*α* can impinge on the insulin/IGF-1 signaling via direct interaction between the IKK complexes and IRS-1. Alternatively, TNF*α*-induced activation of JNK may play a role, as shown by the observation that the downregulation of the IGF-1-dependent signaling exerted by the cytokine does not occur in the presence of a JNK inhibitor (reviewed in [61]). Finally, proinflammatory cytokines may modulate anabolism also by inducing leucine-resistance, resulting in decreased mTOR phosphorylation and reduced protein synthesis [62].

## 4. Adult Myogenesis: Satellite Cells and Adult Stem Cells

In addition to modulations of protein synthesis and breakdown rates, several reports in the last years suggest that also alterations of the myogenic response may play a role in the maintenance of skeletal muscle mass in the adult, in both physiological and pathological states. Myogenesis is the process that guarantees the generation of myoblasts to give rise to skeletal muscle tissue. Embryonic myogenesis is definitely better understood thanks to the extensive work of developmental biologists that generate important genetic tools to establish the exact hierarchical activations of skeletal muscle transcription factors triggering the early embryonic process. In the adulthood, the situation is more complicated since those genetic tools are not sufficient to identify the major key players involved in adult myogenesis. Skeletal muscle injuries are extremely common and mainly caused by intensive muscle exercise, trauma, laceration, burns, freezing, and toxin exposure (the latter are also commonly used in experimental models of muscle regeneration). These insults result in muscle injury that determines a diffuse degeneration followed by the induction of regeneration. However, the characteristics of regeneration have been shown to differ according to the type of injury; thus a direct comparison of the results obtained in various studies is extremely difficult.

When skeletal muscle is damaged, muscular fiber degeneration is compensated by the regeneration of new fibers formed at the expense of resident myogenic satellite cells localized underneath the basal lamina of muscle fibers [63]. Each degeneration process is followed by a new regenerative cycle. Skeletal muscle regeneration is mainly sustained by SCs [64, 65] and this is critical for chronic muscle diseases including muscular dystrophies. In MDs, dystrophic SCs share the same molecular defect and the newly formed fibers during regeneration cycle are susceptible to degeneration. With time, the reservoir of satellite cells is totally consumed and the muscle tissue is progressively replaced by connective tissue. In the adulthood, skeletal muscle represents half of the body weight, and SCs are able to maintain their functionality thanks to the generation of muscle precursor cells able to proliferate and, upon fusion, to generate new fibers. All trunk and limb muscle source cells are originated from embryonic somite source, with exception of the head muscle [66]. Pax3 precursors were identified in the embryonic dorsal aorta; they are able to give rise to both smooth and skeletal muscle cells, suggesting a common origin in the two muscle lineages [67]. Oxidative-slow muscles contain a relatively high number of SCs, up to six times more than fast-glycolytic muscles [68]. SCs can be easily isolated after enzymatic digestion and/or physical trituration [69] or by FACS for specific surface markers including CXCR4, *β*1-integrin, Sca-1, M-cadherin, Syndecan-4, Notch-1, and NCAM/CD56 [70–72]. It is relevant to note that SC myogenic potential seems to be diverse according to the markers considered for cell sorting, thus revealing heterogeneity in SC populations. Pax7 is a transcription factor considered as a biomarker for quiescent and proliferating SCs [73], while Jagged-1 is considered a marker of activated SCs [74]. The expression of Pax7, NCAM, and c-Met has been shown also in human SCs. Numb is an inhibitor of the Notch signaling but also a cell fate determinant and was found asymmetrically distributed in a SC subpopulation, suggesting that only a small subset of SCs retains the stem cell characteristic and undergoes asymmetric division [75]. There are still pending questions regarding SC isolation and characterization. Although encouraging results have been obtained in preclinical [76, 77] and clinical [78] studies, the use of SCs for the treatment of muscle degeneration is hampered by the inability of SCs to pass the endothelial barrier when injected systemically. Further work is needed to confirm and improve the therapeutic efficiency of SC autologous injection for skeletal muscle degeneration.

Bone marrow cells, including MSCs, blood, and muscle-derived CD133^+^ and SP cells, have been also implicated in skeletal muscle regeneration [79]. Several studies have demonstrated that MSCs are incorporated into regenerating skeletal muscle fibers. However, in some cases, engrafted cells failed to express skeletal muscle proteins, suggesting that under standard conditions they fuse rather than differentiate to skeletal muscle potency. In other cases, results have been more encouraging. In bone marrow and peripheral blood, stem cells characterized by the expression of the CD133 antigen are present and have been shown to give rise to dystrophin-positive fibers following their intramuscular transplantation [79].

SP cells are referred to as a small subpopulation of stem cells able to exclude Hoechst 33342 dye and participate to adult myogenesis [80]. Related to the SP populations are the CD34^+^/CD45^−^ cells, known as Sk-34 cells that are apparently derived from CD34^−^/CD45^−^, named Sk-DN cells [81]. Being different from the other myogenic stem cells, they still retain the ability to differentiate into vascular cells, including pericyte, endothelial cell, and smooth muscle cells and peripheral nerve cells as Schwann and perineurial cells [82]. Interstitial Cajal-like cells or telocytes are recently discovered c-Kit cells type populating the muscle interstitium [83]. These cells own a small body (9–15 um) and a certain number of telopodes organized in network to maintain tissue homeostasis and renewal through exosome delivery.

In the interstitium among the fibers are usually present several cell types that were also showed to contribute to adult myogenesis. Whether these cells are missing or altered in pathological muscles and their origins are still heavily debated. These cell types include FAPs, Tcf/L2^+^ cells, and Pw1^+^ cells. Emerging evidences highlight that intramuscular adipocytes and fibrocytes are the differentiated stages of FAPs [84–86]. FAPs are isolated as CD34^+^/Sca-1^+^ [84] or as Sca-1^+^/CD140a^+^ [85] and they are able to differentiate into myoblasts. These cells mediate the ability of HDAC inhibitors to promote skeletal muscle regeneration in mdx mice, animal model for DMD [86]. The key cytokines and growth factors responsible for their paracrine positive effects are still under investigation, and more information regarding the human counterparts is necessary for translational clinical implications. In murine models were identified a particular class of fibroblasts expressing the transcription factor 7-like 2 (Tcf/L2 or Tcf4, [87]). These Tcf4^+^ fibroblasts present in the connective tissue seem also to regulate muscle fiber generation and as such they could be directly related to FAPs. Pw1 is a zinc-finger-containing transcription factor expressed in myoblasts, and it seems as an important marker for myogenic progenitor cells since postnatal muscle growth is severely impaired in mice lacking Pw1 in myogenic lineages [88].

Muscle-derived stem cells are also identified in the skeletal muscle interstitium based on the expression of Flk1, Sca-1, and desmin. Since they are able to differentiate into myogenic, adipogenic, osteogenic, chondrogenic [89], and even hematopoietic [90] lineages, they are strictly associated with mesoangioblasts and Pw1 positive cells although comparative studies are still missing.

Noncanonical progenitors of mesodermal tissues were originally isolated from murine dorsal aorta and for their multipotent characteristic to give rise to mesodermal cell types* in vitro* and* in vivo* they were mesoangioblasts [91]. MABs express CD34/c-Kit/Flk-1 but are negative for NKX2.5/Myf5/Oct4 [92] and are able to give rise to multiple mesodermal lineages* in vitro* and* in vivo* [93]. In the adult muscles, MABs are usually isolated and cloned using their pericyte markers, alkaline phosphatase, Sca-1, NG2 proteoglycan, CD140a, and CD140b [94–99]. MABs are able to differentiate into myogenic, osteogenic, chondrogenic, and adipogenic lineages [97, 98]. Also human MABs display pericyte markers, as CD146/CD140b1/NG2, but are negative for hematopoietic or SC markers CD45^−^/CD34^−^/CD56^−^/CD144^−^/Pax7^−^ [96, 100]. Since a few markers are shared between human pericytes and MSCs (CD10/CD13/CD44/CD73/CD90), the origin and the interaction between those multipotent stem cells are still matter of debate [100, 101]. Interestingly, a comparison among MSCs, MABs, and multipotent adult progenitor cells resulted in specific differention/functional properties that can be partially converted* in vitro* by culture conditions [102]. In addition, Notch signaling seems to have a primary role in modulating the myogenic potential of MSCs and MABs [99].

Stem cell biology in adult myogenesis is a very active and fast moving field, and probably some redundancies in stem cell type and function observed here will be explained with further comparative studies. There is a clear need for more basic research to better understand the interconnected roles of stem cells in skeletal muscle regeneration and to explore the integration of signaling pathways such as Notch and BMP. This new information not only will improve our understanding of the adult myogenesis process but also in principle could reveal potential therapies for the enhancement of tissue repair in acute and chronic skeletal muscle degenerations.

## 5. Inflammation Interferes with the Myogenic Program

**Figure 2:**
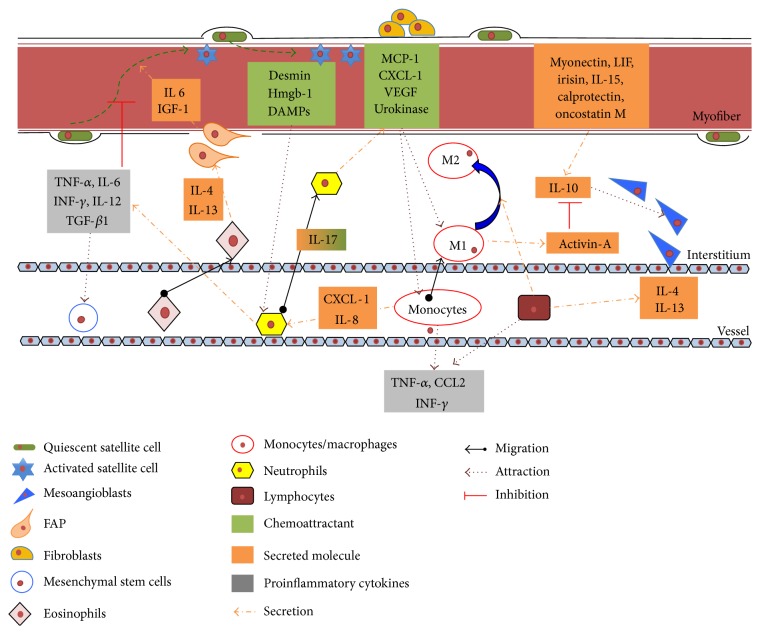
Secreted molecules and paracrine effects from resident and circulating cells involved in skeletal muscle inflammation.

Changes in cellular composition of muscle microenvironment are crucial for metabolic modifications occurring during both acute damage with consequent muscle regeneration and chronic degeneration. Overall, the pathophysiological alterations occurring during the onset and the progression of muscle diseases highlight the complexity of the possible interactions among muscle resident/recruited cells and immunological mediators (Figure 2). The modulation that takes place during acute events, such as muscle trauma inducing regeneration after temporary atrophic conditions, is different from the one occurring during chronic events, such as long lasting inflammatory processes affecting muscle during genetic diseases (MDs or myopathies), cancer-induced muscle atrophy, and sarcopenia.

The inflammatory process during trauma or fractures is a controlled and finely regulated event that through different and defined stages can ensure a complete and efficient reconstruction of muscle fibers. The process begins with the release of chemoattractant molecules like desmin [103] but also heat shock proteins and HMGB1 among many others, responsible for local activation of the innate immune response and comprehensively recognized as damage associated molecular patterns [104]. Muscle injury is usually followed by a local increase in myeloperoxidase activity that reflects neutrophils activation. Their contribution to skeletal muscle regeneration and myofiber remodeling relies on the oxidative and proteolytic modification of damaged tissue, to allow phagocytosis of cellular and matrix debris [105]. Interestingly, targeted ablation of CD11b^+^ cells (including neutrophils and monocytes/macrophages) reduces muscle fiber repair after muscle injury [106]. Moreover, neutrophils release proinflammatory cytokines such as TNF-*α*, IL-1*β*, IFN-*γ*, TGF-*β*1, and IL-12 that reach a peak of concentration 24 h after injury [107] and can modulate the regenerative process in skeletal muscle [108]. Recent findings show that IL-4 production mainly due to eosinophils could play a role during early stages of muscle regeneration [109]. Coherently, muscle cells lacking IL-4 or its receptor show impaired myotube formation [110].

Myogenic cells attract monocytes from the blood stream to their location next to capillaries [111]. Indeed, this privileged position could be the reason for easy access and activation through chemotactic molecules such as MCP-1, macrophage-derived chemokine, fractalkine (CXCL1), VEGF, and the urokinase system [112]. However,* in vivo* studies have reported that monocytes/macrophages recruited into skeletal muscle after injury could come from the connective tissue surrounding muscle/epimysium, where these cells accumulate before invading muscle tissue in the site of damage.

Notably, already twenty years ago, some studies reported that the activation of M1 macrophages (CD68^high^/CD163^−^) at days 1-2 after injury was concomitant to activation and proliferation of SCs. By contrast, M2 macrophages (CD68^low^/CD163^+^) reach the peak of concentration closely to the surface of regenerating myofibers just 4 days after injury, when muscle differentiation is starting [113]. Consistently, different* in vitro* studies showed that SCs increase their proliferation rates when cocultured with M1 macrophages [114], while they enhance both their fusion index and myogenin expression in the presence of M2 cells [106]. Muscle regeneration in mice induced by cardiotoxin injection was impaired after treatment with neutralizing antibodies against Macrophage Colony Stimulating Factor, a cytokine able to activate macrophages [115]. M1 to M2 transition, timing, and correct sequence have been demonstrated to be essential for both resolution of inflammation and myofiber repair. Indeed, IL-10 administration after muscle injury leads to M1 disruption and M2 promotion, resulting in reduced myofiber growth and regeneration [116]. Ablation of IL-10* in vivo* can disturb the transition from M1 to M2 macrophages and decrease fiber growth. When cocultures of muscle cells with macrophages activated through IL-10 to the M2 phenotype are stimulated to proliferate, no effect on MyoD or myogenin expression is observed, showing that M2 macrophages promote the early, proliferative stage of myogenesis [117]. Anyway, the window where IL-10 can promote regeneration is short and if not respected can induce a delay in myogenic program [116, 118]. Moreover, macrophage-derived IL-10 seems as well able to promote skeletal muscle differentiation of MABs, vessel derived myogenic precursors, and IL-10 antibodies can effectively decrease the capabilities of these cells to participate in myofiber commitment [118].

As IL-10, other mediators such as TNF-*α* and IFN-*γ* are produced in the muscle microenvironment and play a role during muscle myogenesis. As an example, TNF-*α* can impair regeneration, keeping SCs in the proliferation stage and inhibiting differentiation, mainly by acting on NF-*κ*B and MyoD [119, 120]. However, a second important role for TNF-*α* is related to myofiber release of activin-A through both NF-*κ*B and p38 MAPK, activating the phosphorylation of SMAD2/3 to inhibit SC differentiation and fusion [121]. M1 macrophages produce high levels of activin-A that,* in vitro*, can act both on the further polarization of M1 macrophages and on the inhibition of anti-inflammatory IL-10 release from M2 cells [122]. Suppression of p38 MAPK signaling is usually followed by Th2 cytokine elevation (IL-10 and TGF-*β*), suggesting the switch in macrophage phenotype during skeletal muscles regeneration [116].

The mechanisms through which IFN-*γ* can directly influence muscle regeneration are still poorly understood and, so far, the knowledge is limited to some* in vitro* data, reporting a delay in proliferation, impaired fusion, and low expression of terminally differentiation markers when this cytokine is added to myoblasts cultures [123–125]. All of these data confirm the vision of skeletal muscle as an endocrine organ able to secrete specific myokines endowed with both paracrine and endocrine functions [126]. Indeed, leukemia inhibitory factor [127] and IL-15 [128] are usually mentioned among the paracrine effectors, while myokines, such as myonectin [129], IL-6 [130], irisin [131], calprotectin [132], and oncostatin M [133], once secreted from muscle tissue, especially during contraction but also in pathologic conditions, are able to induce anti-inflammatory cytokines (IL-1 receptor antagonist and IL-10 [134]).

Despite a transient and finely tuned activation of the inflammatory system required to sustain muscle repair after injury, chronic inflammation can be deleterious, driving uncontrolled wound healing and fibrosis, as well as triggering muscle wasting. For these reasons, systemic administration of immune-modulators can reveal beneficial effects, particularly in the case of anti-inflammatory drugs able to reduce M1-macrophages, as shown, for example, in DMD patients [135]. Interestingly, treatment with cyclosporin A, well-known immunosuppressant drug, was found to have multiple beneficial effects on the myopathic and mitochondrial phenotype of collagen VI a1-null mice, recovering muscle strength and, recently also, increasing Pax7^+^ pool and stimulating the formation of new myofibers [136–138]. This effect may be exerted through an indirect regulation of the inflammatory state that occurs during muscle regeneration [139]. All of these findings have been as well supported by the increased muscle regeneration observed in Ullrich patients undergoing CsA treatment, pointing on one side to immunosuppressive drugs as potent inducer of myogenesis and on the other side underlining the involvement of immune system with myogenesis, at least for these myopathies. Administration of neutralizing antibodies against TNF-*α* to* mdx* mice reduced p38-dependent inflammation, increased Pax7^+^ cells, and impaired the growth of regenerating fibers [140], suggesting the occurrence of an epigenetic link among inflammation, activated p38 MAPK, and Pax7 expression from SCs during regeneration [140].

Further studies are needed to understand the function of SCs and their possible induction to differentiate as a means to counteract muscle atrophy. Increased Pax7 and decreased myogenin levels have been reported also in the cachectic muscle of mice bearing the C26 carcinoma and in cancer patients, opening the possibility that cancer-driven inflammation induces muscle atrophy, dysregulating SC differentiation program [52, 141, 142]. SC accumulation was demonstrated to be due to an increase in the ERK MAPK signaling able to maintain the cells in an undifferentiated state [141]. Treatment with a chemical inhibitor of ERK phosphorylation can indeed rescue the levels of Pax7 and myogenin [141]. Lately, this process of SC accumulation has been pointed as contributing to muscle atrophy and has been related to NF-*κ*B, known transcription factor able to be activated by many proinflammatory cytokines. In the skeletal muscle of cachectic mice, NF-*κ*B is able to sustain Pax7 expression [52], and moreover, in the same study, the authors revealed that other muscle precursors, such as MSCs, participate in muscle wasting since they cannot complete their myogenic commitment. Chronic injury and proinflammatory cytokines due to tumor progression could be responsible for differentiation program failure.

When mesenchymal progenitors are missing or are unable to display their prodifferentiation effects on SCs, these latter ones actually contribute to fibrosis in mdx mice, leading to pathogenic effects in MDs [143]. In the last years, cells expressing CD34/Sca1/PDGFRa and not related with other lineages, such as hematopoietic, endothelial, or skeletal muscle, have received more and more attention. Indeed, these cells can both differentiate* in vitro* and* in vivo* towards fibroblast and adipocytes producing aSMA and perilipin, respectively [84, 143]. Recently, suppression of fibroadipogenic phenotype of mesenchymal cells through HDAC inhibitors has been demonstrated to induce the myogenic transcriptional activity in young mice by upregulating MyoD. On the contrary, FAPs taken from old mice fail in the activation of promyogenic phenotype mainly because of HDAC inhibitor resistant [144]. These last results already suppose a different behavior of the microenvironment that is actually acting on the myogenic program of SCs in the elderly. Aging of skeletal muscle should not be underestimated when considering myogenic potential. Indeed, geriatric SCs show reduced proliferation and differentiation potential and can easily switch from a quiescent to a senescent state [145]. Some studies have already focused on the importance of the microenvironment where SCs are studied, since if old murine SCs are exposed to a young environment or growth factors, their ability to proliferate and differentiate is partly restored, suggesting the extreme importance the environment has on the single cells and on the other side the plasticity cells can have [146]. During aging, extrinsic factors can alter SC functions, starting from the niche where they lay, since fibrous connective tissue is usually increasing [147, 148]. In addition, SCs in the elderly were shown to convert to a fibrogenic lineage mainly due to humoral factors. In particular, this lineage conversion seems to depend on Wnt signaling [149]. Lately, a comparison between old and young SCs identified an increased expression in JAK-STAT pathway with aging that could avoid their differentiation in old conditions [150]. This pathway is of particular importance in chronic inflammatory conditions, since infiltration of inflammatory cells and increased circulating levels of proinflammatory cytokines (such as TNF-*α*) can have together a detrimental effect on skeletal muscle regeneration [151]. Moreover, this pathway has usually a pivotal role in transduction of extracellular signals from cytokines and growth factors with particular importance for inflammatory cells [152]. Interestingly, in a recent work, murine and human SCs were demonstrated to progress in their differentiation thanks to STAT3 and a fine regulation of the activation of this protein could interfere with myogenesis [153]. The authors indeed speculate that chronic degenerative stimuli could favor this prodifferentiative pathway leading to exhaustion of the SC pool. Indeed, in chronic regenerative conditions, a pharmacologic inhibition of STAT3 could have a therapeutic relevance.

## 6. Conclusions

Inflammation is a common trait of several pathological conditions characterized by the loss of muscle mass. During the past, a direct link was established among proinflammatory cytokines, modulation of intracellular signaling pathways, and protein breakdown. In recent years, an additional hypothesis, suggesting the impairment of the myogenic program as underlying cause of muscle atrophy, is becoming popular. In this line, the importance of a finely orchestrated balance between pro- and anti-inflammatory cytokines in regulating physiological myogenesis is a well-established concept, while data suggesting the relevance of inflammation in impaired myogenesis are growing. Inflammation likely contributes to muscle depletion by both enhancing protein breakdown and impairing myogenesis in parallel and no priority between the two processes can actually be identified. The other way round, an effective strategy aimed at counteracting muscle wasting should take into consideration not only anabolic/catabolic aspects but analogously the continuous involvement of the myogenic counterpart. In addition, the role of interstitial and circulating progenitors involved in myogenesis and paracrine effects is also critical to modulate inflammatory responses in muscle wasting conditions.

The adoption of anti-inflammatory agents for the treatment of chronic wasting diseases has been widely described [154] and is not the topic of the present review; however, the modulatory effect on inflammation exerted by exercise training deserves a short consideration. Regular, nonstrenuous exercise seems to be protective against inflammation. Indeed, combined endurance and resistance training in elder subjects resulted in decrease of proinflammatory CD14^+^/CD16^+^ monocytes and low levels of TNF-*α* production* in vitro* [155]. Moreover, circulating IL-10 and regulatory T-cells (CD4^+^/CD25^+^/CD127^low^) increase in well-trained athletes with respect to a sedentary age matched population, even in resting conditions [156]. Keeping in mind the above-mentioned anti-inflammatory effect and considering that exercise is the most physiological stimulus able to coordinate both myogenesis and muscle hypertrophy, the adoption of patient-tailored exercise protocols will potentially have impact on muscle wasting associated with distinct pathologies.

## Abbreviations

CHF: Chronic heart failure
CKD: Chronic kidney disease
COPD: Chronic obstructive pulmonary disease
BMP: Bone morphogenetic protein
IGF-1: Insulin-like growth factor-1
GH: Growth hormone
ROS: Reactive oxygen species
mTOR: Mammalian target of rapamycin
TGF: Transforming growth factor
ACTRIIB: Activin receptor type IIB
iNOS: Inducible nitric oxide synthase
HDAC: Histone deacetylase
ATF4: Activating transcription factor 4
TNF-*α*: Tumor necrosis factor alpha
IL: Interleukin
IFN-*γ*: Interferon gamma
TWEAK: TNF-like weak inducer of apoptosis
PGC-1*α*: Peroxisome proliferator-activated receptor coactivator 1 alpha
IRS-1: Insulin receptor substrate-1
MD: Muscular dystrophy
SC: Satellite cell
MPC: Muscle precursor cells
FACS: Fluorescent-activated cell sorting
NCAM: Neuronal cell adhesion molecule
MSC: Mesenchymal stem cell
SP: Side population
FAP: Fibroadipogenic progenitor
DMD: Duchenne muscular dystrophy
MAB: Mesoangioblast
Sca-1: Stem cell antigen-1
HMGB1: High mobility group box 1
MCP-1: Macrophage-derived chemoattractant protein 1
VEGF: Vascular endothelial growth factor
ERK: Extracellular-signal regulated kinase.

## References

[1] Biolo G., Cederholm T., Muscaritoli M. (2014). Muscle contractile and metabolic dysfunction is a common feature of sarcopenia of aging and chronic diseases: from sarcopenic obesity to cachexia. *Clinical Nutrition*.

[2] Muscaritoli M., Anker S. D., Argilés J. (2010). Consensus definition of sarcopenia, cachexia and pre-cachexia: Joint document elaborated by Special Interest Groups (SIG) ‘cachexia-anorexia in chronic wasting diseases’ and ‘ nutrition in geriatrics’. *Clinical Nutrition*.

[3] Fearon K., Strasser F., Anker S. D. (2011). Definition and classification of cancer cachexia: an international consensus. *The Lancet Oncology*.

[4] Harrington D., Anker S. D., Chua T. P. (1997). Skeletal muscle function and its relation to exercise tolerance in chronic heart failure. *Journal of the American College of Cardiology*.

[5] Taskin S., Stumpf V. I., Bachmann J., Weber C., Martignoni M. E., Friedrich O. (2014). Motor protein function in skeletal abdominal muscle of cachectic cancer patients. *Journal of Cellular and Molecular Medicine*.

[6] Llano-Diez M., Renaud G., Andersson M. (2012). Mechanisms underlying ICU muscle wasting and effects of passive mechanical loading. *Critical Care*.

[7] Costelli P., Baccino F. M. (2003). Mechanisms of skeletal muscle depletion in wasting syndromes: Role of ATP-ubiquitin-dependent proteolysis. *Current Opinion in Clinical Nutrition & Metabolic Care*.

[8] Penna F., Baccino F. M., Costelli P. (2014). Coming back: autophagy in cachexia. *Current Opinion in Clinical Nutrition and Metabolic Care*.

[9] Hasselgren P.-O., Wray C., Mammen J. (2002). Molecular regulation of muscle cachexia: it may be more than the proteasome. *Biochemical and Biophysical Research Communications*.

[10] Du J., Wang X., Miereles C. (2004). Activation of caspase-3 is an initial step triggering accelerated muscle proteolysis in catabolic conditions. *Journal of Clinical Investigation*.

[11] Krawiec B. J., Frost R. A., Vary T. C., Jefferson L. S., Lang C. H. (2005). Hindlimb casting decreases muscle mass in part by proteasome-dependent proteolysis but independent of protein synthesis. *The American Journal of Physiology—Endocrinology and Metabolism*.

[12] Masiero E., Agatea L., Mammucari C. (2009). Autophagy is required to maintain muscle mass. *Cell Metabolism*.

[13] Penna F., Costamagna D., Pin F. (2013). Autophagic degradation contributes to muscle wasting in cancer cachexia. *American Journal of Pathology*.

[14] Carnio S., LoVerso F., Baraibar M. A. (2014). Autophagy impairment in muscle induces neuromuscular junction degeneration and precocious aging. *Cell Reports*.

[15] Bodine S. C., Latres E., Baumhueter S. (2001). Identification of ubiquitin ligases required for skeletal Muscle Atrophy. *Science*.

[16] Gomes M. D., Lecker S. H., Jagoe R. T., Navon A., Goldberg A. L. (2001). Atrogin-1, a muscle-specific F-box protein highly expressed during muscle atrophy. *Proceedings of the National Academy of Sciences of the United States of America*.

[17] Kudryashova E., Kudryashov D., Kramerova I., Spencer M. J. (2005). Trim32 is a ubiquitin ligase mutated in limb girdle muscular dystrophy type 2H that binds to skeletal muscle myosin and ubiquitinates actin. *Journal of Molecular Biology*.

[18] Ye J., Zhang Y., Xu J., Zhang Q., Zhu D. (2007). FBXO40, a gene encoding a novel muscle-specific F-box protein, is upregulated in denervation-related muscle atrophy. *Gene*.

[19] Shi J., Luo L., Eash J., Ibebunjo C., Glass D. J. (2011). The SCF-Fbxo40 complex induces IRS1 ubiquitination in skeletal muscle, limiting IGF1 signaling. *Developmental Cell*.

[20] Sartori R., Schirwis E., Blaauw B. (2013). BMP signaling controls muscle mass. *Nature Genetics*.

[21] Vinciguerra M., Hede M., Rosenthal N. (2010). Comments on point:counterpoint: IGF is/is not the major physiological regulator of muscle mass. IGF-1 is a major regulator of muscle mass during growth but not for adult myofiber hypertrophy. *Journal of Applied Physiology*.

[22] Castets P., Rüegg M. A. (2013). MTORC1 determines autophagy through ULK1 regulation in skeletal muscle. *Autophagy*.

[23] Sandri M., Sandri C., Gilbert A. (2004). Foxo transcription factors induce the atrophy-related ubiquitin ligase atrogin-1 and cause skeletal muscle atrophy. *Cell*.

[24] Mammucari C., Milan G., Romanello V. (2007). FoxO3 Controls Autophagy in Skeletal Muscle In Vivo. *Cell Metabolism*.

[25] Lokireddy S., McFarlane C., Ge X. (2011). Myostatin induces degradation of sarcomeric proteins through a Smad3 signaling mechanism during skeletal muscle wasting. *Molecular Endocrinology*.

[26] Trendelenburg A. U., Meyer A., Rohner D., Boyle J., Hatakeyama S., Glass D. J. (2009). Myostatin reduces Akt/TORC1/p70S6K signaling, inhibiting myoblast differentiation and myotube size. *The American Journal of Physiology—Cell Physiology*.

[27] Moresi V., Williams A. H., Meadows E. (2010). Myogenin and class II HDACs control neurogenic muscle atrophy by inducing E3 ubiquitin ligases. *Cell*.

[28] Ebert S. M., Monteys A. M., Fox D. K. (2010). The transcription factor ATF4 promotes skeletal myofiber atrophy during fasting. *Molecular Endocrinology*.

[29] Fox D. K., Ebert S. M., Bongers K. S. (2014). p53 and ATF4 mediate distinct and additive pathways to skeletal muscle atrophy during limb immobilization. *The American Journal of Physiology: Endocrinology and Metabolism*.

[30] Li J. P., Lu L., Wang L. J., Zhang F. R., Shen W. F. (2011). Increased serum levels of S100B are related to the severity of cardiac dysfunction, renal insufficiency and major cardiac events in patients with chronic heart failure. *Clinical Biochemistry*.

[31] Niewczas M. A., Gohda T., Skupien J. (2012). Circulating TNF receptors 1 and 2 predict ESRD in type 2 diabetes. *Journal of the American Society of Nephrology*.

[32] Seruga B., Zhang H., Bernstein L. J., Tannock I. F. (2008). Cytokines and their relationship to the symptoms and outcome of cancer. *Nature Reviews Cancer*.

[33] Walston J., Fedarko N., Yang H. (2008). The physical and biological characterization of a frail mouse model. *Journals of Gerontology—Series A Biological Sciences and Medical Sciences*.

[34] Wang B., Yang G., Liang X., Zhu M., Du M. (2014). Grape seed extract prevents skeletal muscle wasting in interleukin 10 knockout mice. *BMC Complementary and Alternative Medicine*.

[35] Durham W. J., Dillon E. L., Sheffield-Moore M. (2009). Inflammatory burden and amino acid metabolism in cancer cachexia. *Current Opinion in Clinical Nutrition and Metabolic Care*.

[36] Bonetto A., Aydogdu T., Kunzevitzky N. (2011). STAT3 activation in skeletal muscle links muscle wasting and the acute phase response in cancer cachexia. *PLoS ONE*.

[37] Biolo G., Declan Fleming R. Y., Maggi S. P., Nguyen T. T., Herndon D. N., Wolfe R. R. (2002). Inverse regulation of protein turnover and amino acid transport in skeletal muscle of hypercatabolic patients. *Journal of Clinical Endocrinology and Metabolism*.

[38] Klaude M., Mori M., Tjäder I., Gustafsson T., Wernerman J., Rooyackers O. (2012). Protein metabolism and gene expression in skeletal muscle of critically ill patients with sepsis. *Clinical Science*.

[39] Johns N., Stephens N. A., Preston T. (2012). Muscle protein kinetics in cancer cachexia. *Current Opinion in Supportive and Palliative Care*.

[40] Tisdale M. J. (2008). Catabolic mediators of cancer cachexia. *Current Opinion in Supportive and Palliative Care*.

[41] Matthys P., Heremans H., Opdenakker G., Billiau A. (1991). Anti-interferon-gamma antibody treatment, growth of Lewis lung tumours in mice and tumour-associated cachexia. *European Journal of Cancer and Clinical Oncology*.

[42] Costelli P., Carbo N., Tessitore L. (1993). Tumor necrosis factor-*α* mediates changes in tissue protein turnover in a rat cancer cachexia model. *The Journal of Clinical Investigation*.

[43] Strassmann G., Fong M., Freter C. E., Windsor S., D'Alessandro F., Nordan R. P. (1993). Suramin interferes with interleukin-6 receptor binding in vitro and inhibits colon-26-mediated experimental cancer cachexia in vivo. *Journal of Clinical Investigation*.

[44] Mangili A., Murman D. H., Zampini A. M., Wanke C. A. (2006). Nutrition and HIV infection: review of weight loss and wasting in the era of highly active antiretroviral therapy from the nutrition for healthy living cohort. *Clinical Infectious Diseases*.

[45] Molanouri Shamsi M., Hassan Z. H., Gharakhanlou R. (2014). Expression of interleukin-15 and inflammatory cytokines in skeletal muscles of STZ-induced diabetic rats: effect of resistance exercise training. *Endocrine*.

[46] Lee J. S. W., Auyeung T.-W., Kwok T., Lau E. M. C., Leung P.-C., Woo J. (2007). Associated factors and health impact of sarcopenia in older Chinese men and women: a cross-sectional study. *Gerontology*.

[47] Reed S. A., LaVigne E. K., Jones A. K., Patterson D. F., Schauer A. L. The aging horse: effects of inflammation on muscle satellite cells.

[48] Cai D., Frantz J. D., Tawa N. E. (2004). IKKbeta/NF-kappaB activation causes severe muscle wasting in mice. *Cell*.

[49] Di Marco S., Mazroui R., Dallaire P. (2005). NF-*κ*B-mediated MyoD decay during muscle wasting requires nitric oxide synthase mRNA stabilization, HuR protein, and nitric oxide release. *Molecular and Cellular Biology*.

[50] Rhoads M. G., Kandarian S. C., Pacelli F., Doglietto G. B., Bossola M. (2010). Expression of NF-*κ*B and I*κ*B proteins in skeletal muscle of gastric cancer patients. *European Journal of Cancer*.

[51] Op den Kamp C. M., Langen R. C., Snepvangers F. J. (2013). Nuclear transcription factor kappa B activation and protein turnover adaptations in skeletal muscle of patients with progressive stages of lung cancer cachexia. *The American Journal of Clinical Nutrition*.

[52] He W. A., Berardi E., Cardillo V. M. (2013). NF-*κ*B-mediated Pax7 dysregulation in the muscle microenvironment promotes cancer cachexia. *The Journal of Clinical Investigation*.

[53] Paul P. K., Gupta S. K., Bhatnagar S. (2010). Targeted ablation of TRAF6 inhibits skeletal muscle wasting in mice. *The Journal of Cell Biology*.

[54] Mittal A., Bhatnagar S., Kumar A. (2010). The TWEAK-Fn14 system is a critical regulator of denervation-induced skeletal muscle atrophy in mice. *Journal of Cell Biology*.

[55] Hindi S. M., Mishra V., Bhatnagar S. (2014). Regulatory circuitry of TWEAK-Fn14 system and PGC-1alpha in skeletal muscle atrophy program. *The FASEB Journal*.

[56] Frost R. A., Lang C. H. (2008). Regulation of muscle growth by pathogen-associated molecules. *Journal of Animal Science*.

[57] Crossland H., Constantin-Teodosiu D., Gardiner S. M., Constantin D., Greenhaff P. L. (2008). A potential role for Akt/FOXO signalling in both protein loss and the impairment of muscle carbohydrate oxidation during sepsis in rodent skeletal muscle. *Journal of Physiology*.

[58] Liu Y., Chen F., Odle J. (2013). Fish oil increases muscle protein mass and modulates akt/FOXO, TLR4, and NOD signaling in weanling piglets after lipopolysaccharide challenge. *Journal of Nutrition*.

[59] Wang D. T., Yin Y., Yang Y. J. (2014). Resveratrol prevents TNF-alpha-induced muscle atrophy via regulation of Akt/mTOR/FoxO1 signaling in C2C12 myotubes. *International Immunopharmacology*.

[60] White J. P., Puppa M. J., Gao S., Sato S., Welle S. L., Carson J. A. (2013). Muscle mTORC1 suppression by IL-6 during cancer cachexia: a role for AMPK. *American Journal of Physiology: Endocrinology and Metabolism*.

[61] Grounds M. D., Radley H. G., Gebski B. L., Bogoyevitch M. A., Shavlakadze T. (2008). Implications of cross-talk between tumour necrosis factor and insulin-like growth factor-1 signalling in skeletal muscle. *Clinical and Experimental Pharmacology and Physiology*.

[62] Mcintire K. L., Chen Y., Sood S., Rabkin R. (2014). Acute uremia suppresses leucine-induced signal transduction in skeletal muscle. *Kidney International*.

[63] Mauro A. (1961). Satellite cell of skeletal muscle fibers. *The Journal of Biophysical and Biochemical Cytology*.

[64] Quattrocelli M., Cassano M., Crippa S., Perini I., Sampaolesi M. (2010). Cell therapy strategies and improvements for muscular dystrophy. *Cell Death and Differentiation*.

[65] Berardi E., Annibali D., Cassano M., Crippa S., Sampaolesi M. (2014). Molecular and cell-based therapies for muscle degenerations: a road under construction. *Frontiers in Physiology*.

[66] Scaal M., Christ B. (2004). Formation and differentiation of the avian dermomyotome. *Anatomy and Embryology*.

[67] Esner M., Meilhac S. M., Relaix F., Nicolas J.-F., Cossu G., Buckingham M. E. (2006). Smooth muscle of the dorsal aorta shares a common clonal origin with skeletal muscle of the myotome. *Development*.

[68] Yin H., Price F., Rudnicki M. A. (2013). Satellite cells and the muscle stem cell niche. *Physiological Reviews*.

[69] Musarò A., Barberi L. (2010). Isolation and culture of mouse satellite cells. *Methods in Molecular Biology*.

[70] Conboy M. J., Cerletti M., Wagers A. J., Conboy I. M. (2010). Immuno-analysis and FACS sorting of adult muscle fiber-associated stem/precursor cells. *Methods in Molecular Biology*.

[71] Chapman M. R., Balakrishnan K. R., Li J. (2013). Sorting single satellite cells from individual myofibers reveals heterogeneity in cell-surface markers and myogenic capacity. *Integrative Biology*.

[72] Castiglioni A., Hettmer S., Lynes M. D. (2014). Isolation of progenitors that exhibit myogenic/osteogenic bipotency in vitro by fluorescence-activated cell sorting from human fetal muscle. *Stem Cell Reports*.

[73] Seale P., Sabourin L. A., Girgis-Gabardo A., Mansouri A., Gruss P., Rudnicki M. A. (2000). Pax7 is required for the specification of myogenic satellite cells. *Cell*.

[74] Gnocchi V. F., White R. B., Ono Y., Ellis J. A., Zammit P. S. (2009). Further characterisation of the molecular signature of quiescent and activated mouse muscle satellite cells. *PLoS ONE*.

[75] Shinin V., Gayraud-Morel B., Gomès D., Tajbakhsh S. (2006). Asymmetric division and cosegregation of template DNA strands in adult muscle satellite cells. *Nature Cell Biology*.

[76] Partridge T. A., Morgan J. E., Coulton G. R., Hoffman E. P., Kunkel L. M. (1989). Conversion of mdx myofibres from dystrophin-negative to -positive by injection of normal myoblasts. *Nature*.

[77] Mouly V., Aamiri A., Périé S. (2005). Myoblast transfer therapy: is there any light at the end of the tunnel?. *Acta Myologica*.

[78] Skuk D., Roy B., Goulet M. (2004). Dystrophin expression in myofibers of Duchenne muscular dystrophy patients following intramuscular injections of normal myogenic cells. *Molecular Therapy*.

[79] Cossu G., Sampaolesi M. (2007). New therapies for Duchenne muscular dystrophy: challenges, prospects and clinical trials. *Trends in Molecular Medicine*.

[80] Motohashi N., Asakura A. (2012). Molecular regulation of muscle satellite cell self-renewal. *Journal of Stem Cell Research & Therapy*.

[81] Tamaki T., Uchiyama Y., Akatsuka A. (2010). Plasticity and physiological role of stem cells derived from skeletal muscle interstitium: contribution to muscle fiber hyperplasia and therapeutic use. *Current Pharmaceutical Design*.

[82] Tamaki T., Okada Y., Uchiyama Y. (2007). Synchronized reconstitution of muscle fibers, peripheral nerves and blood vessels by murine skeletal muscle-derived CD34(−)/45 (−) cells. *Histochemistry and Cell Biology*.

[83] Cretoiu S. M., Popescu L. M. (2014). Telocytes revisited. *Biomolecular Concepts*.

[84] Joe A. W. B., Yi L., Natarajan A. (2010). Muscle injury activates resident fibro/adipogenic progenitors that facilitate myogenesis. *Nature Cell Biology*.

[85] Uezumi A., Fukada S.-I., Yamamoto N., Takeda S., Tsuchida K. (2010). Mesenchymal progenitors distinct from satellite cells contribute to ectopic fat cell formation in skeletal muscle. *Nature Cell Biology*.

[86] Mozzetta C., Consalvi S., Saccone V. (2013). Fibroadipogenic progenitors mediate the ability of HDAC inhibitors to promote regeneration in dystrophic muscles of young, but not old Mdx mice. *EMBO Molecular Medicine*.

[87] Mathew S. J., Hansen J. M., Merrell A. J. (2011). Connective tissue fibroblasts and Tcf4 regulate myogenesis. *Development*.

[88] Mitchell K. J., Pannérec A., Cadot B. (2010). Identification and characterization of a non-satellite cell muscle resident progenitor during postnatal development. *Nature Cell Biology*.

[89] Peng H., Huard J. (2004). Muscle-derived stem cells for musculoskeletal tissue regeneration and repair. *Transplant Immunology*.

[90] Zou K., Huntsman H. D., Valero M. C. (2015). Mesenchymal stem cells augment the adaptive response to eccentric exercise. *Medicine & Science in Sports & Exercise*.

[91] Cossu G., Bianco P. (2003). Mesoangioblasts—vascular progenitors for extravascular mesodermal tissues. *Current Opinion in Genetics and Development*.

[92] De Angelis L., Berghella L., Coletta M. (1999). Skeletal myogenic progenitors originating from embryonic dorsal aorta coexpress endothelial and myogenic markers and contribute to postnatal muscle growth and regeneration. *Journal of Cell Biology*.

[93] Minasi M. G., Riminucci M., De Angelis L. (2002). The meso-angioblast: a multipotent, self-renewing cell that originates from the dorsal aorta and differentiates into most mesodermal tissues. *Development*.

[94] Sampaolesi M., Torrente Y., Innocenzi A. (2003). Cell therapy of *α*-sarcoglycan null dystrophic mice through intra-arterial delivery of mesoangioblasts. *Science*.

[95] Sampaolesi M., Blot S., D'Antona G. (2006). Mesoangioblast stem cells ameliorate muscle function in dystrophic dogs. *Nature*.

[96] Dellavalle A., Sampaolesi M., Tonlorenzi R. (2007). Pericytes of human skeletal muscle are myogenic precursors distinct from satellite cells. *Nature Cell Biology*.

[97] Tonlorenzi R., Dellavalle A., Schnapp E., Cossu G., Sampaolesi M. (2007). Isolation and characterization of mesoangioblasts from mouse, dog, and human tissues. *Current Protocols in Stem Cell Biology*.

[98] Quattrocelli M., Palazzolo G., Perini I., Crippa S., Cassano M., Sampaolesi M. (2012). Mouse and human mesoangioblasts: isolation and characterization from adult skeletal muscles. *Methods in Molecular Biology*.

[99] Quattrocelli M., Costamagna D., Giacomazzi G., Camps J., Sampaolesi M. (2014). Notch signaling regulates myogenic regenerative capacity of murine and human mesoangioblasts. *Cell Death and Disease*.

[100] Crisan M., Yap S., Casteilla L. (2008). A perivascular origin for mesenchymal stem cells in multiple human organs. *Cell Stem Cell*.

[101] Caplan A. I. (2008). All MSCs are pericytes?. *Cell Stem Cell*.

[102] Roobrouck V. D., Clavel C., Jacobs S. A. (2011). Differentiation potential of human postnatal mesenchymal stem cells, mesoangioblasts, and multipotent adult progenitor cells reflected in their transcriptome and partially influenced by the culture conditions. *Stem Cells*.

[103] Linder E., Lehto V. P., Stenman S. (1979). Activation of complement by cytoskeletal intermediate filaments. *Nature*.

[104] Rubartelli A., Lotze M. T. (2007). Inside, outside, upside down: damage-associated molecular-pattern molecules (DAMPs) and redox. *Trends in Immunology*.

[105] Nguyen H. X., Lusis A. J., Tidball J. G. (2005). Null mutation of myeloperoxidase in mice prevents mechanical activation of neutrophil lysis of muscle cell membranes in vitro and in vivo. *The Journal of Physiology*.

[106] Arnold L., Henry A., Poron F. (2007). Inflammatory monocytes recruited after skeletal muscle injury switch into antiinflammatory macrophages to support myogenesis. *The Journal of Experimental Medicine*.

[107] Hawke T. J., Garry D. J. (2001). Myogenic satellite cells: physiology to molecular biology. *Journal of Applied Physiology*.

[108] Warren G. L., Hulderman T., Jensen N. (2002). Physiological role of tumor necrosis factor alpha in traumatic muscle injury. *The FASEB Journal*.

[109] Heredia J. E., Mukundan L., Chen F. M. (2013). Type 2 innate signals stimulate fibro/adipogenic progenitors to facilitate muscle regeneration. *Cell*.

[110] Horsley V., Jansen K. M., Mills S. T., Pavlath G. K. (2003). IL-4 acts as a myoblast recruitment factor during mammalian muscle growth. *Cell*.

[111] Chiristov C., Chrétien F., Abou-Khalil R. (2007). Muscle satellite cells and endothelial cells: close neighbors and privileged partners. *Molecular Biology of the Cell*.

[112] Chazaud B., Sonnet C., Lafuste P. (2003). Satellite cells attract monocytes and use macrophages as a support to escape apoptosis and enhance muscle growth. *The Journal of Cell Biology*.

[113] St. Pierre B. A., Tidball J. G. (1994). Differential response of macrophage subpopulations to soleus muscle reloading after rat hindlimb suspension. *Journal of Applied Physiology*.

[114] Bencze M., Negroni E., Vallese D. (2012). Proinflammatory macrophages enhance the regenerative capacity of human myoblasts by modifying their kinetics of proliferation and differentiation. *Molecular Therapy*.

[115] Segawa M., Fukada S.-I., Yamamoto Y. (2008). Suppression of macrophage functions impairs skeletal muscle regeneration with severe fibrosis. *Experimental Cell Research*.

[116] Perdiguero E., Sousa-Victor P., Ruiz-Bonilla V. (2011). p38/MKP-1-regulated AKT coordinates macrophage transitions and resolution of inflammation during tissue repair. *The Journal of Cell Biology*.

[117] Deng B., Wen J., Ding Y. (2012). Functional analysis of pig myostatin gene promoter with some adipogenesis- and myogenesis-related factors. *Molecular and Cellular Biochemistry*.

[118] Bosurgi L., Corna G., Vezzoli M. (2012). Transplanted mesoangioblasts require macrophage IL-10 for survival in a mouse model of muscle injury. *The Journal of Immunology*.

[119] Bakkar N., Wang J., Ladner K. J. (2008). IKK/NF-*κ*B regulates skeletal myogenesis via a signaling switch to inhibit differentiation and promote mitochondrial biogenesis. *Journal of Cell Biology*.

[120] Guttridge D. C., Mayo M. W., Madrid L. V., Wang C.-Y., Baldwin A. S. J. (2000). NF-kappaB-induced loss of MyoD messenger RNA: possible role in muscle decay and cachexia. *Science*.

[121] Trendelenburg A. U., Meyer A., Jacobi C., Feige J. N., Glass D. J. (2012). TAK-1/p38/nNF*κ*B signaling inhibits myoblast differentiation by increasing levels of Activin A. *Skeletal Muscle*.

[122] Sierra-Filardi E., Puig-Kröger A., Blanco F. J. (2011). Activin A skews macrophage polarization by promoting a proinflammatory phenotype and inhibiting the acquisition of anti-inflammatory macrophage markers. *Blood*.

[123] Kalovidouris A. E., Plotkin Z., Graesser D. (1993). Interferon-*γ* inhibits proliferation, differentiation, and creatine kinase activity of cultured human muscle cells. II. A possible role in myositis. *Journal of Rheumatology*.

[124] Villalta S. A., Deng B., Rinaldi C., Wehling-Henricks M., Tidball J. G. (2011). IFN-*γ* promotes muscle damage in the mdx mouse model of duchenne muscular dystrophy by suppressing M2 macrophage activation and inhibiting muscle cell proliferation. *Journal of Immunology*.

[125] Londhe P., Davie J. K. (2011). Gamma interferon modulates myogenesis through the major histocompatibility complex class II transactivator, CIITA. *Molecular and Cellular Biology*.

[126] Mathur N., Pedersen B. K. (2008). Exercise as a mean to control low-grade systemic inflammation. *Mediators of Inflammation*.

[127] Broholm C., Mortensen O. H., Nielsen S. (2008). Exercise induces expression of leukaemia inhibitory factor in human skeletal muscle. *The Journal of Physiology*.

[128] Quinn L. S., Anderson B. G., Strait-Bodey L., Stroud A. M., Argués J. M. (2009). Oversecretion of interleukin-15 from skeletal muscle reduces adiposity. *The American Journal of Physiology—Endocrinology and Metabolism*.

[129] Seldin M. M., Wong G. W. (2014). Regulation of tissue crosstalk by skeletal muscle-derived myonectin and other myokines. *Adipocyte*.

[130] Pedersen B. K. (2012). Muscular interleukin-6 and its role as an energy sensor. *Medicine & Science in Sports & Exercise*.

[131] Boström P., Wu J., Jedrychowski M. P. (2012). A PGC1-alpha-dependent myokine that drives brown-fat-like development of white fat and thermogenesis. *Nature*.

[132] Mortensen O. H., Andersen K., Fischer C. (2008). Calprotectin is released from human skeletal muscle tissue during exercise. *The Journal of Physiology*.

[133] Pedersen L., Olsen C. H., Pedersen B. K., Hojman P. (2012). Muscle-derived expression of the chemokine CXCL1 attenuates diet-induced obesity and improves fatty acid oxidation in the muscle. *The American Journal of Physiology—Endocrinology and Metabolism*.

[134] Steensberg A., Fischer C. P., Keller C., Møller K., Pedersen B. K. (2003). IL-6 enhances plasma IL-1ra, IL-10, and cortisol in humans. *American Journal of Physiology—Endocrinology and Metabolism*.

[135] Hodgetts S., Radley H., Davies M., Grounds M. D. (2006). Reduced necrosis of dystrophic muscle by depletion of host neutrophils, or blocking TNFalpha function with Etanercept in mdx mice. *Neuromuscular Disorders*.

[136] Irwin W. A., Bergamin N., Sabatelli P. (2003). Mitochondrial dysfunction and apoptosis in myopathic mice with collagen VI deficiency. *Nature Genetics*.

[137] Grumati P., Coletto L., Sabatelli P. (2010). Autophagy is defective in collagen VI muscular dystrophies, and its reactivation rescues myofiber degeneration. *Nature Medicine*.

[138] Gattazzo F., Molon S., Morbidoni V. (2014). Cyclosporin a promotes in vivo myogenic response in collagen VI-deficient myopathic mice. *Frontiers in Aging Neuroscience*.

[139] Serrano A. L., Mann C. J., Vidal B., Ardite E., Perdiguero E., Muñoz-Cánoves P. (2011). Cellular and molecular mechanisms regulating fibrosis in skeletal muscle repair and disease. *Current Topics in Developmental Biology*.

[140] Palacios D., Mozzetta C., Consalvi S. (2010). TNF/p38*α*/polycomb signaling to Pax7 locus in satellite cells links inflammation to the epigenetic control of muscle regeneration. *Cell Stem Cell*.

[141] Penna F., Costamagna D., Fanzani A., Bonelli G., Baccino F. M., Costelli P. (2010). Muscle wasting and impaired myogenesis in tumor bearing mice are prevented by ERK inhibition. *PLoS ONE*.

[142] Ramamoorthy S., Donohue M., Buck M. (2009). Decreased Jun-D and myogenin expression in muscle wasting of human cachexia. *American Journal of Physiology—Endocrinology and Metabolism*.

[143] Uezumi A., Ito T., Morikawa D. (2011). Fibrosis and adipogenesis originate from a common mesenchymal progenitor in skeletal muscle. *Journal of Cell Science*.

[144] Saccone V., Consalvi S., Giordani L. (2014). HDAC-regulated myomiRs control BAF60 variant exchange and direct the functional phenotype of fibro-adipogenic progenitors in dystrophic muscles. *Genes & Development*.

[145] Sousa-Victor P., Gutarra S., García-Prat L. (2014). Geriatric muscle stem cells switch reversible quiescence into senescence. *Nature*.

[146] Carlson B. M., Faulkner J. A. (1989). Muscle transplantation between young and old rats: Age of host determines recovery. *The American Journal of Physiology—Cell Physiology*.

[147] Goldspink G., Fernandes K., Williams P. E., Wells D. J. (1994). Age-related changes in collagen gene expression in the muscles of mdx dystrophic and normal mice. *Neuromuscular Disorders*.

[148] Barberi L., Scicchitano B. M., de Rossi M. (2013). Age-dependent alteration in muscle regeneration: the critical role of tissue niche. *Biogerontology*.

[149] Brack A. S., Conboy M. J., Roy S. (2007). Increased Wnt signaling during aging alters muscle stem cell fate and increases fibrosis. *Science*.

[150] Price F. D., von Maltzahn J., Bentzinger C. F. (2014). Inhibition of JAK-STAT signaling stimulates adult satellite cell function. *Nature Medicine*.

[151] Jang T.-S., Lee E.-J., Jo J.-H. (2012). Fibrous membrane of nano-hybrid poly-L-lactic acid/silica xerogel for guided bone regeneration. *Journal of Biomedical Materials Research Part B: Applied Biomaterials*.

[152] Harrison D. A. (2012). The JAK/STAT pathway. *Cold Spring Harbor Perspectives in Biology*.

[153] Tierney M. T., Aydogdu T., Sala D. (2014). STAT3 signaling controls satellite cell expansion and skeletal muscle repair. *Nature Medicine*.

[154] Fearon K., Arends J., Baracos V. (2013). Understanding the mechanisms and treatment options in cancer cachexia. *Nature Reviews Clinical Oncology*.

[155] Timmerman K. L., Flynn M. G., Coen P. M., Markofski M. M., Pence B. D. (2008). Exercise training-induced lowering of inflammatory (CD14^+^CD16^+^) monocytes: a role in the anti-inflammatory influence of exercise?. *Journal of Leukocyte Biology*.

[156] Handzlik M. K., Shaw A. J., Dungey M., Bishop N. C., Gleeson M. (2013). The influence of exercise training status on antigen-stimulated IL-10 production in whole blood culture and numbers of circulating regulatory T cells. *European Journal of Applied Physiology*.

